# Recognizing cisplatin as a potential radiation recall trigger: case report and focused systematic review

**DOI:** 10.1007/s00066-023-02059-9

**Published:** 2023-03-15

**Authors:** Bálint Tamaskovics, Jan Haussmann, Kimia Karimi, Michael Daum-Marzian, Peter Arne Gerber, Felix Knapp, Kathrin Scheckenbach, Edwin Bölke, Christiane Matuschek, Wilfried Budach

**Affiliations:** 1 Department of Radiation Oncology, Medical Faculty and University Hospital Düsseldorf, Heinrich Heine University, Moorenstr. 5, 40225 Düsseldorf, Germany; 2 Department of Particle Therapy, University Hospital Essen, West German Proton Therapy Centre Essen (WPE), West German Cancer Centre (WTZ), Essen, Germany; 3 Department of Radiation Oncology, Helios Hospital Krefeld, Krefeld, Germany; 4 Department of Dermatology, Medical Faculty and University Hospital Düsseldorf, Heinrich Heine University, Düsseldorf, Germany; 5 Department of Otorhinolaryngology and Head and Neck Surgery, Medical Faculty and University Hospital Düsseldorf, Heinrich Heine University, Düsseldorf, Germany

**Keywords:** Radiation recall phenomenon, RRD, CDDP, Dermatitis, Multidrug combination

## Abstract

We present a case of mild radiation recall dermatitis triggered by cisplatin chemotherapy given simultaneously to re-irradiation. The dermatitis area correlated to skin exposure of the previous radiation therapy, characterizing the reaction clearly as a recall. Cisplatin has not yet been recognized as a potential trigger for recall reactions. Although it was part of several reported multidrug trigger combinations, all review works referred to cisplatin as not suspicious, suggesting the combination partner as the effector. We performed a focused systematic literature review aiming to re-evaluate the real role of cisplatin as a (co-)triggering factor. In total, 30 reported cases were found, 90% triggered by multidrug combinations. The latter tended to cause more severe symptoms. Besides findings supporting the 20 Gy-threshold theory, no correlation between radiation dose and severity or prevalence was found. Recognition of cisplatin as a trigger of the recall phenomenon and its supportive management may prevent unnecessary cessation of systemic chemotherapy. Systematic reporting of recall events as a secondary endpoint of prospective clinical trials applying radiation therapy could support understanding the recall phenomenon.

**Supplementary Information:**

The online version of this article (10.1007/s00066-023-02059-9) contains supplementary material, which is available to authorized users.

## Introduction

Cisplatin—also called cisplatinum, CDDP, and cis-diamminedichloroplatinum(II)—is a well-established cytotoxic agent with several therapeutic indications, prescribed as monotherapy as well as part of various combinations with other chemotherapeutics and/or with radiation therapy (RT). Due to its widespread use, oncologists should be aware of rare adverse events, such as the chance of triggering radiation recall.

The radiation recall phenomenon is a rare, unpredictable acute inflammatory reaction at a previously irradiated area. Radiation recall dermatitis (RRD) is the most commonly reported manifestation, likely because of the clearly visible symptoms. In addition, sporadic cases of radiation recall pneumonitis (RRP), myositis, mucositis, esophagitis, colitis, and proctitis are known [1]. Onset is usually within days to weeks after the trigger event, which can be intravenous, subcutaneous, or oral administration of a drug [2–4], a vaccine [5, 6], or herbal products [7], but also heat [8] or cold [9] exposition. The incidence varies between 1.8 and 11.5% according to the few available systematic reports, although low numbers of included patient and retrospective design limit the usability of these data [10].

Until now, cisplatin has not been recognized as a trigger factor, not even in most recent reviews [1, 11–14]. CDDP was the only one among relatively frequently applied drugs in a cohort which did not cause a recall phenomenon [15]. In the few cases in which cisplatin was part of the combination triggering a recall, the concomitant drugs have always been suspected of being the trigger.

We designed and performed a systematic review of the literature to identify and re-evaluate all potential cisplatin-induced and co-triggered radiation recall reactions (RRR).

## Case presentation

**Fig. 1:**
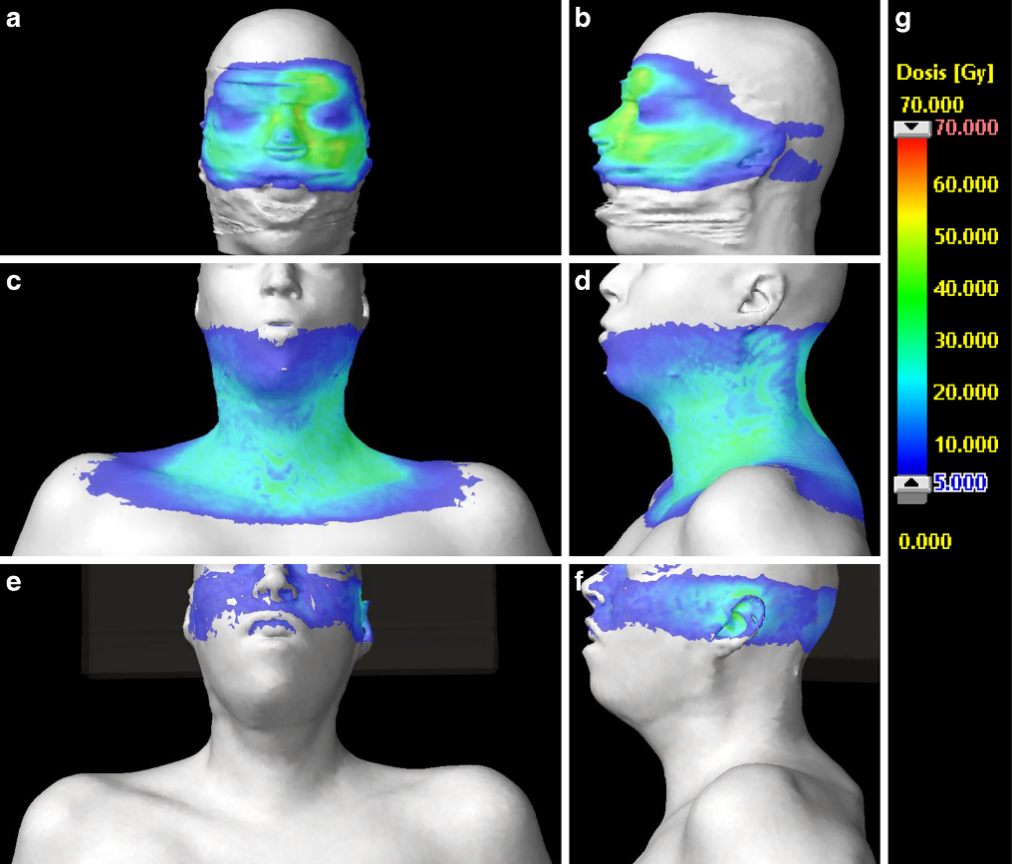
Surface dose of the first radiation treatment plan in front view (**a**) and side view (**b**). Surface dose of the previous radiation treatment plan in front view (**c**) and side view (**d**). Surface dose of the current radiation treatment plan in front view (**e**) and side view (**f**). Lowest shown dose is 5 Gy in *deep blue* color, while 20 Gy is *light blue*, as shown in the corresponding color-dose scale (**g**)

A 38-year-old Caucasian woman presented in our academic head and neck cancer center with bilateral cervical lymphadenopathy persisting for 2 years after resection of an in situ adenocarcinoma of the left nasal cavity and the left paranasal sinuses with subsequent local radiotherapy (50.4/1.8 Gy, IMRT, Fig. 1a, b). Bilateral neck dissection with subsequent radiotherapy (66 Gy/2 Gy, IMAT, Fig. 1c, d) was performed because of metastases of a high-grade adenocarcinoma in multiple lymph nodes with extracapsular extension. Two cycles of cisplatin (20 mg/m^2^ and day, d1–5) and 5‑fluorouracil (600 mg/m^2^ and day, d1–5, continuous infusion) were given parallel to radiation therapy. Typical radiodermatitis (CTCAE grade 1), mucositis (CTCAE grade 2), and odyno-/dysphagia (CTCAE grade 2) developed and resolved completely within 4 weeks after the end of radiation therapy.

A year later, the patient presented with a locally advanced recurrence of an adenocarcinoma (G3) in the left middle ear, Eustachian tube, and nasopharynx, without any distal metastases. A combined transpetrosal and endonasal radical tumor resection of the middle ear, tube, and nasopharynx via Fisch type C access with middle ear obliteration with abdominal fat and a free muscle transplant as seal for the resected Eustachian tube canal and an external auditory canal closure was performed. Postoperative re-irradiation (60 Gy/2 Gy, IMAT, Fig. 1e, f) in combination with weekly cisplatin (40 mg/m^2^) was initiated, as high-dose, curatively intended re-irradiation is a standard approach of our site, reported to be safe and beneficial regarding the outcome [16–19].

**Fig. 2:**
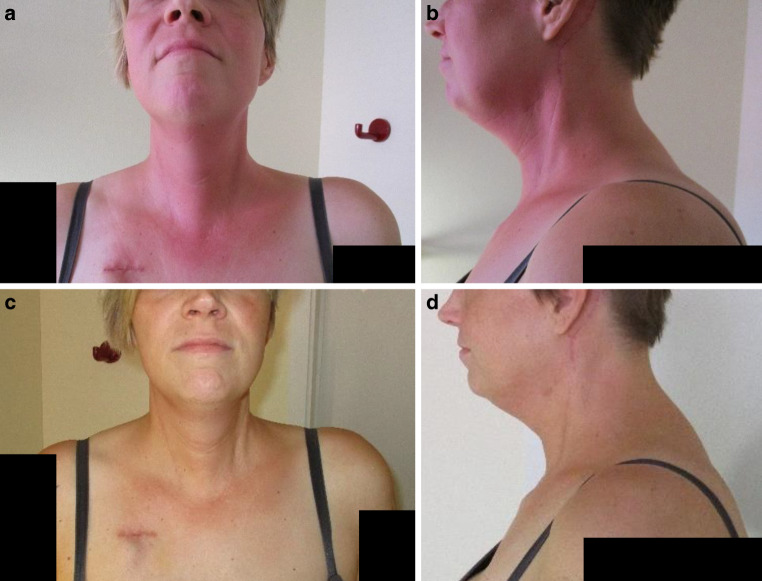
Moderate erythema 1 day after the infusion of cisplatin in front (**a**) and side views (**b**). Mild hyperpigmentation and subsided erythema 3 days later in front (**c**) and side views (**d**)

Six hours after the first administration of chemotherapy, an erythema of the neck (CTCAE grade 1) with dysesthesia (CTCAE grade 1) occurred, as shown in Fig. 2a, b. The sharp border of the inflammatory skin reaction corresponded to the bilateral cervical lymph node irradiation site a year before, roughly in accordance with the 20-Gy isodose at the skin surface. No other potential trigger than cisplatin could be identified. The recall reaction resolved within 3 days under topical therapy with panthenol and alpha-linolenic acid containing moisturizing creams (Fig. 2c, d). The treatment protocol was not affected. Mild RRD recurred after each of the five subsequent cisplatin infusions, with decrescendo kinetics.

## Review methods

### Data sources and search strategy

The Preferred Reporting Items for Systematic Reviews and Meta-Analyses (PRISMA) statement was used to guide the conduct and reporting of this review [20] (Supplementary Table S1). The review protocol was registered in PROSPERO, the international prospective register of systematic reviews [21]. We searched three major online databases (PubMed, https://pubmed.ncbi.nlm.nih.gov; Web of Science, https://www.webofscience.com; and Scopus, https://www.scopus.com) until 31 October 2022 for ([″RRD″ OR ″recall″ OR ″radiation recall″ OR ″recall dermatitis″ OR ″recall phenomenon″] AND [″CDDP″ OR ″cisplatin″ OR ″cisplatinum″]) with no limitations. In addition, we searched reference lists of the relevant review articles with Elsevier’s Scopus.

### Case selection

All reported radiation recall reaction cases with cisplatin as a potential trigger were included. Two reviewers (BT and KK) independently evaluated the titles and abstracts for these inclusion criteria. For each potentially eligible case, two reviewers assessed the full text. In cases of disagreement, a third reviewer (JH) was involved, and decisions could have been taken by consensus.

### Data extraction

We used a predesigned data extraction form to collect relevant information. According to Camidge and Price [12], the dose of radiation and drug(s), time from end of radiotherapy to recall-triggering drug exposure, time to developing RRR and its grade, therapeutic interventions, the time to resolution, and the effect of any drug rechallenge were extracted from case reports. Furthermore, we assessed radiation therapy characteristics, patient and tumor characteristics, and also previous chemotherapy lines, when reported. We calculated the equivalent dose at 2 Gy (EQD2) with the linear quadratic model. An alpha/beta ratio of 3 Gy was assumed to estimate the long-term radiation effect at the site of the recall phenomenon. Missing values were marked as not reported in the summary table. We excluded no cases because of missing data, and included all cases stated as a recall event by the original authors.

## Results

The search strategy identified 241 references, 63 were removed as duplicates. After the initial screening based on titles and abstracts, the full texts of 88 articles and one congress abstract were retrieved for further evaluation. Besides our case, 29 further cisplatin-induced recall cases (18 reports) are included in this review. The PRISMA flowchart (Supplementary Figure S1) and characteristics table with all 30 cases (Supplementary Table S2) can be accessed in the online supplement.

### Cisplatin as single trigger of radiation recall

Three reported recall reactions triggered by cisplatin monotherapy were found, including our case [22, 23]. Besides two cases of mild dermatitis in the head and neck region, a moderate case occurred in the pelvic area. Onset time from trigger infusion ranged between a few minutes and several hours. Radiation dose, when known, was high (66–70 Gy in 2‑Gy fractions), time interval from RT to RRD ranged between 3 and 12 months. Details on rechallenge and therapy are reported for two of the single-drug cases. Both patients were successfully rechallenged, the therapeutic regime was not affected by the RRD. In one case, no recurrence of the recall phenomenon was observed, while in the other case, a decrescendo type of recurrence of the mild dermatitis was seen. In both cases, topical supportive therapy had been prescribed: corticosteroids and antihistamines in the first case, and panthenol with alpha-linolenic acid in the second (Table 1).

**Table 1:** Characteristics of all reported cisplatin-induced radiation recall reactions

	Smith 2002 [22]	Mandal 2021 [23]	Present case
Age (years)	64	21	39
Sex	Male	Female	Female
Recall reaction	Dermatitis	Dermatitis	Dermatitis
Severity (CTCAE grade)	Moderate (2)	Mild (1)	Mild (1)
Body site	Pelvis	Face	Face, neck
Time to onset recall reaction after implementing cisplatin	Not reported	Few minutes	6 h
Rechallenge	Not reported	Yes	Yes
Recurrence of RRD after rechallenge	Not reported	No recurrence of the phenomenon	Recurred after each of the five subsequent treatments
Effect on treatment protocol	Not reported	Delayed (10 days)	Not modified
Supportive therapy	Not reported	Topical corticosteroids and antihistaminic	Topical panthenol, alpha-linolenic acid
Radiation technique	Not reported	IMRT	IMAT
Target dose/fraction dose	Not reported	70 Gy/2 Gy	66 Gy/2 Gy
Calculated late effect (α/β = 3) EQD2 (Gy) in the area of the recall phenomenon	Not reported	70.0 Gy	66.0 Gy
Time interval between end of radiation to start trigger drug	12 months	3 months	12 months
Primary tumor	Bladder cancer	Nasopharyngeal carcinoma	Sinonasal adenocarcinoma
Previous chemotherapy regimen(s)	Not reported	2 × cisplatin/5-FU + weekly cisplatin	2 × cisplatin/5-FU

*CTCAE* Common Terminology Criteria for Adverse Events, *EQD2* equivalent dose at 2 Gy, *IMRT* intensity-modulated radiation therapy, *IMAT* intensity-modulated arc therapy, *RRD* radiation recall dermatitis

### Cisplatin in multidrug trigger combinations

**Fig. 3:**
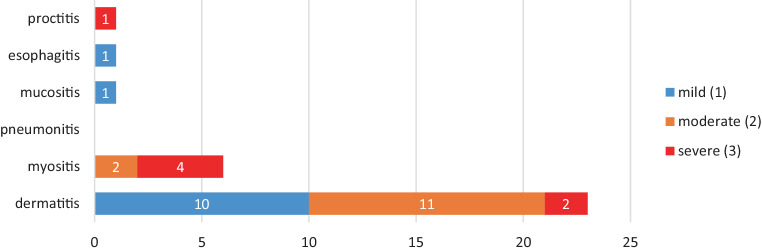
Recall reaction types and severity in single- and multidrug regimens containing cisplatin

In the majority of the reported RRR cases, cisplatin was part of the multidrug trigger combination. Usually, the substances other than cisplatin were identified as the trigger. As shown in Table 2, the most frequent co-triggers were antimetabolites (gemcitabine [24–30], 5‑FU [31–34], pemetrexed [34–36], and cytarabine [34]), while other cytotoxic agents with known high recall potential such as taxanes [37, 38], anthracyclines [22, 34], and vinorelbine [39] were also present. Seven severe recall reactions occurred, four cases of myositis, two of dermatitis and one of proctitis. Co-trigger drugs in these cases were gemcitabine, pemetrexed, and 5‑FU—the latter two induced dermatitis (Fig. 3).

**Table 2:** Radiation recall reaction characteristics, single- and multidrug regimens containing cisplatin

Drug regimen	Number of reported cases	Radiation recall reactions	Severity (CTCAE) median (range)	Body site(s)	Mean EQD2 dose (range)	Time range RT to trigger	Time range trigger to RRR
Cisplatin	3	Dermatitis	1 (1–2)	Face, neck, trunk	68 Gy (66–70)	3–12 months	A few minutes–6 hours
Gemcitabine/cisplatin	7	Dermatitis, myositis, proctitis	3 (2–3)	Neck, trunk, shoulder	43 Gy (18–56)	1 months–37 years	1 weeks–3 months
Cisplatin/5-fluorouracil	6	Dermatitis, myositis, mucositis	1 (1–3)	Scalp, oral, neck, trunk, perianal	64 Gy (43–74)	3 weeks–9 years	3 days–3 months
Cisplatin/pemetrexed	4	Dermatitis	2 (1–3)	Scalp, trunk	42 Gy (N.A.)	3–4 weeks	2–5 days
Cisplatin/cytarabine ± rituximab	2	Dermatitis, esophagitis	1 (1)	Thorax	N.A.	3–4 weeks	3–5 days
Paclitaxel/cisplatin	3	Dermatitis	1 (1)	Breast, trunk	56 Gy (50–67)	4–8 months	3 hours–5 days
Cisplatin/doxorubicin ± cyclophosphamide	2	Dermatitis	1 (1–2)	Trunk	N.A.	3 weeks–9 months	3–5 days
Cisplatin/epirubicin ± mitomycin	2	Dermatitis	2 (2)	Head	N.A.	3–26 months	N.A.
Cisplatin/vinorelbine	1	Dermatitis	2 (2)	Trunk	73 Gy (N.A.)	1 week	2 weeks

*N.A.* not available, *CTCAE* Common Terminology Criteria for Adverse Events, *EQD2* equivalent dose at 2 Gy, *RT* radiation therapy, *RRR* radiation recall reaction, *Gy* Gray

### Radiotherapy dose and technique

Two reports (with five cases) did not provide any data on radiotherapy dose and technique. Solely the total target dose was reported for the largest case series of eight RRR, although a standard fractionation scheme can be assumed in three of them. Radiation technique was not described for two further cases.

In two cases, the previous exposure to ionizing radiation was not due to classical radiation therapy: one patient was subject to extraordinary occupational sun exposure as a sailor [31], while one other patient had been treated with whole-body narrow-band ultraviolet B (NB-UVB) irradiation [28]. A dose calculation in these latter two cases was not feasible.

Most of the cases were conventionally or moderately hypofractionated, besides three palliative high-fraction-dose irradiations. None of the patients had been treated using high-precision high-dose techniques. Two-dimensional anteroposterior-posteroanterior (AP-PA) or tangential opposed fields were used in six of the cases, three-dimensional conformal RT (3D-CRT) in four of the cases, while IMRT and IMAT were used in one case each.

The calculated mean late effect EQD2 (α/β = 3 Gy) was 54.3 Gy (standard deviation: 15.3 Gy; range: 17.6–74.4 Gy). There was no correlation between dose and the severity of the recall reaction. Due to the small sample size, no correlation between radiation technique and the degree of severity could be determined.

## Discussion

Smith et al. reported the very first case of cisplatin as a standalone trigger in a pathological case series in 2002 [22]. Perhaps because of the incomplete details, or for other reasons, reviewers have not recognized this case since then. Consequently, cisplatin has generally been discussed as a less suspicious part of the known trigger drug combinations.

Recently, Mandal et al. reported a case of radiation recall dermatitis triggered by a chemotherapy combination of cisplatin and 5‑fluorouracil [23]. The reaction occurred during the cisplatin infusion before the initiation of 5‑FU, thus cisplatin is clearly a single-drug trigger in this case.

Our case, together with the two previously mentioned, clearly supports the finding of cisplatin being a potential single-drug trigger of RRD. All three reactions were mild to moderate, and were successfully treated with topical supportive therapy (including corticosteroids) with short resolution periods. RDD did not affect the actual chemotherapy regimen.

The role of cisplatin as a component of multidrug combinations is to be re-evaluated. Since the current demonstration of cisplatin being a potential recall trigger, it is no longer feasible to declare only the combination partner drug as suspect. However, taxanes, anthracyclines, and most of the antimetabolites are known to cause a recall reaction more frequently [14, 15, 40, 41].

The median severity was higher in combinations with gemcitabine, pemetrexed, epirubicin, and vinorelbine (Table 2). Most of the grade 2–3 reactions lead to a delay in or discontinuation of current chemotherapy, and systemic steroids and/or antihistamines had to be applied. Dose-limiting toxicity was generally more common than in cases with cisplatin as a single-drug trigger.

Radiation recall pneumonitis is a potentially lethal reaction, regularly diagnosed and reported in severe stages [42]. Some asymptomatic, grade 1 cases are diagnosed incidentally. Taxanes cause recall pneumonitis relatively frequently [43]. On the contrary, we found no cisplatin (co-)induced RRP cases reported as yet. In the age of immunotherapy, the role of pneumonitis is emerging, thus awareness of RRP should also be increased [44, 45].

Radiation recall dermatitis nearly always affects the radiation port if a 3D conformal or 2D technic was used. With the emergence of rotational intensity-modulated radiation therapy (IMRT), the field borders are not rectangular anymore, while skin and underlying subcutaneous connective tissues receive lower dose to a relatively larger volume [46], referred as a “dose bath.” The corresponding skin doses could be visualized at the surface [47]. A 20-Gy skin dose has been suggested as the recall threshold by several authors [48, 49], although 40 Gy has also been discussed for bleomycin-triggered RRD [50]. For better comparability, we calculated EQD2 for the cisplatin-involved recall cases. Our result does not contradict the 20 Gy-threshold theory according to the observed 2σ range of 23.7–84.9 Gy EQD2. RRD remains, however, unpredictable, without any further clear dose correlation of the prevalence and the severity.

Regarding prevalence, no clear data are available. Cisplatin was the only frequent drug in a cohort trial, which did not cause a recall phenomenon [15]. Because of mild symptoms and the quick recovery seen in the three known cases, some cisplatin-induced recall cases may have remained below the detection limit. The prospective registering of (potential) radiation recall toxicities in all phase II, III, and IV clinical trials with medium or long-term follow-up would be essential to determine prevalence. Since releasing version 4.0 of the Common Terminology Criteria for Adverse Events (CTCAE), radiation recall dermatitis has been recognized as an independent event [51]. On the contrary, radiation recall pneumonitis, myositis, mucositis, etc. did not get their own categories, and can therefore only be reported generally as pneumonitis, myositis, etc. Table 3 presents the actual CTCAE (version 5.0) classifications to be used for reporting RRR in trials [52]. The severity definitions for RRD do not differ from those for radiation dermatitis. Adding radiation recall events, at least RRP and recall myositis, at the next planned update of the CTCAE catalogue would provide the opportunity for more systematic recognition and reporting of RRR events in the context of prospective clinical trials, ultimately allowing for a better prevalence estimation.

**Table 3:** Recall-relevant Common Terminology Criteria for Adverse Events (CTCAE) version 5.0

Term	Grade 1	Grade 2	Grade 3	Grade 4	Grade 5
Radiation recall reaction (dermatologic)	Faint erythema or dry desquamation	Moderate to brisk erythema; patchy moist desquamation, mostly confined to skin folds and creases; moderate edema	Moist desquamation in areas other than skin folds and creases; bleeding induced by minor trauma or abrasion	Life-threatening consequences; skin necrosis or ulceration of full thickness dermis; spontaneous bleeding from involved site; skin graft indicated	Death
Dermatitis radiation	Faint erythema or dry desquamation	Moderate to brisk erythema; patchy moist desquamation, mostly confined to skin folds and creases; moderate edema	Moist desquamation in areas other than skin folds and creases; bleeding induced by minor trauma or abrasion	Life-threatening consequences; skin necrosis or ulceration of full thickness dermis; spontaneous bleeding from involved site; skin graft indicated	Death
Pneumonitis	Asymptomatic; clinical or diagnostic observations only; intervention not indicated	Symptomatic; medical intervention indicated; limiting instrumental ADL	Severe symptoms; limiting self-care ADL; oxygen indicated	Life-threatening respiratory compromise; urgent intervention indicated (e.g., tracheotomy or intubation)	Death
Myositis	Mild pain	Moderate pain associated with weakness; pain limiting instrumental ADL	Pain associated with severe weakness; limiting self-care ADL	Life-threatening consequences; urgent intervention indicated	–
Esophagitis	Asymptomatic; clinical or diagnostic observations only; intervention not indicated	Symptomatic; altered eating/swallowing; oral supplements indicated	Severely altered eating/swallowing; tube feeding, TPN, or hospitalization indicated	Life-threatening consequences; urgent operative intervention indicated	Death
Proctitis	Rectal discomfort, intervention not indicated	Symptomatic (e.g., rectal discomfort, passing blood or mucus); medical intervention indicated; limiting instrumental ADL	Severe symptoms; fecal urgency or stool incontinence; limiting self-care ADL	Life-threatening consequences; urgent intervention indicated	Death

*ADL* activity of daily living, *TPN* total parenteral nutrition

Interesting is the fact that two of the three cases with RRR triggered by cisplatin as a single trigger were young and female. According to the literature, there is no evidence for the existence of a special patient population with an increased risk for RRR. Within the whole group (cisplatin as part of a trigger combination), none of the sexes are under or overrepresented (59% male and 41% female). However, all patients younger than 50 years were female, and median female age was 45 years, in contrast to the median male age of 64 years. On the contrary, the characteristics of all patients treated with any cisplatin-based chemotherapy regime are not known, leading to a rather incalculable prevalence. Further data should be collected to prove whether young women have a higher risk for a cisplatin-triggered radiation recall reaction.

The recall phenomenon typically develops within several days after initiation of the inducing agent. In four of the found cases, the onset time of the recall reaction was more than 20 days from the trigger event, although due to the symptom kinetics and circumstances, there is no doubt in classifying them as RRR. On the other hand, one RRD reported by Melnyk et al. was considered as idiopathic by the authors, while the reaction was seen 9 months after cisplatin therapy [53].

Spugnini et al. reported a feline RRD case triggered by electrochemotherapy using cisplatin [54]. This approach uses direct cisplatin injection into the tumor and surrounding tissues followed by square or biphasic electric pulses to increase uptake of the drug by the cancer cells. Thus, rather similar to cisplatin extravasation induced tissue injury, as in the other case reported by Bairey et al. [55]. In our opinion, these latter cases are less relevant for RRR, in agreement with Shapiro et al. [56].

A relatively large proportion of the screened RRR cases (in 38 of 81 reports) were related to cisplatin only in terms of medical history, i.e., given long before the recall event. This finding could, however, be well explained by the widespread oncologic use of this alkylating agent.

## Conclusion

The reported case reminds us to increase oncology caregivers’ awareness that a radiation recall reaction might be induced by cisplatin—a drug not considered as a potential recall trigger before. Recognition of the phenomenon may prevent unnecessary cessation of systemic chemotherapy.

The review highlights the commonly mild clinical presentation and the option of rechallenge in most of the cases. Besides verifying the theory of a 20 Gy dose threshold for RRD, no other dose correlation could be shown. RRR remains an unpredictable stochastic late toxicity.

In general, increasing the awareness and consequent reporting of all radiation recall reactions is recommended. The integration of screening for radiation recall as an adverse event in future prospective clinical trials could provide refined prevalence data.

## Supplementary Information

**Supplementary Figure S1**: Literature search process (PRISMA 2020 flow diagram)

**Supplementary Table S1**: PRISMA 2020 checklist

**Supplementary Table S2**: Characteristics table of the found 30 cisplatin-(co)induced radiation recall reaction cases

## Abbreviations

2D: Two-dimensional
3D CRT: Three-dimensional conformal radiation therapy
5‑FU: 5‑Fluorouracil
AP-PA: Anteroposterior-posteroanterior
CDDP: Cis-diamminedichloroplatinum(II)
CTCAE: Common Terminology Criteria for Adverse Events
EQD2: Equivalent dose at 2 Gy
IMAT: Intensity-modulated arc therapy
IMRT: Intensity-modulated radiation therapy
NB-UVB: Narrow-band ultraviolet B
PRISMA: Preferred Reporting Items for Systematic Reviews and Meta-Analyses
RRD: Radiation recall dermatitis
RRP: Radiation recall pneumonitis
RRR: Radiation recall reaction
RT: Radiation therapy
SD: Standard deviation
