# Supplementary material for: Recognizing cisplatin as a potential radiation recall trigger: case report and focused systematic review
Source: Strahlenther Onkol. 2023 Mar 15;199(7):611–20. doi: 10.1007/s00066-023-02059-9 (PMC10281908; doi:10.1007/s00066-023-02059-9)
Supplement: Supplementary file 1 — Supplementary Figure S1: Literature search process (PRISMA 2020 flow diagram) [file 66_2023_2059_MOESM1_ESM.docx]

**Identification of studies via other methods**

**Identification of studies via databases and registers**

Duplicate records removed before screening:

(n = 63)

Records identified manually though citation tracking

(n = 12)

*Records identified from*:

PubMed (n = 49)

Scopus (n = 132)

Web of Science (n = 48)

**Identification**

Abstracts screened

(n = 165)

Records excluded for irrelevance based on title and abstract

(n = 87)

Reports not retrieved

(n = 0)

Reports sought for retrieval

(n = 78)

Reports sought for retrieval

(n = 12)

Reports not retrieved

(n = 1)

**Screening**

Reports assessed for eligibility

(n = 78)

Reports assessed for eligibility

(n = 11)

*Reports excluded*:

cisplatin in the discussion,
but no trigger role (n = 3)

review (n = 5)

*Reports excluded*:

cisplatin in the medical history, but no trigger role (n = 38)

discussion or review (n = 15)

irrelevance (n = 9)

non-human (n = 1)

**Included**

Relevant cases included in review

(n = 29)

Reports of included cases

(n = 18)

**Supplementary Figure 1: Literature search process (PRISMA 2020 flow diagram)**

*From:*  Page MJ, McKenzie JE, Bossuyt PM, Boutron I, Hoffmann TC, Mulrow CD, et al. The PRISMA 2020 statement: an updated guideline for reporting systematic reviews. BMJ 2021;372:n71. doi: 10.1136/bmj.n71. For more information, visit: <http://www.prisma-statement.org/>
